# Tic‐Talk: Voices on Tourette's Labelling

**DOI:** 10.1002/mdc3.70110

**Published:** 2025-05-02

**Authors:** Giulia Raffaele, Osman Malik, Seonaid Anderson, Lauren Corcoran, Andreas Hartmann, Davide Martino, Nanette Mol Debes, Kirsten R. Müller‐Vahl, Christelle Nilles, Tamara Pringsheim, Sara Sopena, Natalia Szejko, Owen Tasmin, Kinga K. Tomczak, Tammy Hedderly

**Affiliations:** ^1^ GKT School of Medical Education King's College London London UK; ^2^ Department of Children's Neuroscience Evelina London Children's Hospital, Guys and St Thomas National Health Service Trust and South London and Maudsley National Health Service Trust London UK; ^3^ Neuro‐Diverse.Org Brussels Belgium; ^4^ Evelina London Children's Hospital Guy's and St Thomas Trust and King's College London London UK; ^5^ Centre de Référence National Maladie Rare ‘Syndrome Gilles de la Tourette’, DMU Neurosciences, AP‐HP Sorbonne Université, Groupe Hospitalier Pitié‐Salpêtrière Paris France; ^6^ Department of Clinical Neurosciences University of Calgary Calgary Alberta Canada; ^7^ Hotchkiss Brain Institute University of Calgary Calgary Alberta Canada; ^8^ Department of Pediatrics Copenhagen University Hospital‐Herlev and Gentofte Herlev Denmark; ^9^ Department of Clinical Medicine University of Copenhagen Copenhagen Denmark; ^10^ Department of Psychiatry Social Psychiatry and Psychotherapy, Hannover Medical School Hannover Germany; ^11^ Department of Neurology Hôpital Fondation Rothschild Paris France; ^12^ Department of Bioethics Medical University of Warsaw Warsaw Poland; ^13^ Department of Neurology Boston Children's Hospital, Harvard Medical School Boston Massachusetts USA

**Keywords:** Tourette syndrome, diagnostic labelling, tics, movement disorders, neurodiversity

## Background

Tourette syndrome (TS) is a neurodevelopmental disorder characterized by involuntary motor and vocal tics. Georges Gilles de la Tourette, the first neurologist to describe the condition in 1885, identified 9 cases with motor and vocal tics. However, he concluded that only 4 patients had a true diagnosis of TS, with the others showing anxiety, obsessive‐compulsive disorder (OCD), and other behaviors.^1^, ^2^

Although TS is often diagnosed during childhood, its symptoms peak between the age 8 and 12 years, a period of physical and social development in which patients are potentially affected by stigma, shaping their view of the condition in their later life. One of the main factors contributing to the social stigma related to TS is the misrepresentation of the typical presentation of tics, focusing on a minor cohort of people with TS who have coprolalia. This portrayal has been reinforced by traditional media and, more recently, amplified through social media platforms like TikTok and YouTube.^3^, ^4^, ^5^ These platforms, particularly popular among younger audiences, promote sensational, outrageous, or humorous content to attract views and shares.^6^, ^7^, ^8^ Although such videos often include hashtags like #tourettesyndrome, they predominantly highlight extreme or severe cases. As a result, viewers are led to believe that these rare and exaggerated presentations are typical of TS, perpetuating misconceptions and reinforcing negative stereotypes about the condition.

This has significant implications for individuals living with the condition and their caregivers, particularly affecting areas such as education, social life, and employment.^9^

In our clinical work at Evelina London, many patients voiced concerns about the TS label, citing stigma and its impact on perception. Several families highlighted their preference for different names such as tics, tic spectrum, or tic disorder.

This, alongside a literature review to explore concepts around labeling and stigma in the movement clinics, revealed dissatisfaction around the current language used in TS, and mostly in the wider context of neurodiversity.

In *NeuroTribes*, for example, Silberman criticizes the traditional medical model for its deficit‐centric view, predominantly led by non‐autistic researchers, in contrast to the neurodiversity paradigm. The latter supports the neuro‐affirmative movement, which perceives neurodivergence as a natural genetic variation rather than a deficit, focusing on the experiences and preferences of neurodiverse individuals.^10^, ^11^

In recent years, there has been a growing movement among autism advocates to reframe autism spectrum disorder (ASD) within the framework of neurodiversity, promoting strength‐based language and approaches in assessments and interventions, disagreeing on the use of “syndrome” and “disorder.”^12^

This paradigm shift is particularly relevant among the TS community, as patients are often diagnosed with coexisting attention‐deficit hyperactivity disorder (ADHD), OCD or behaviors (OCD/B), and ASD.^13^

Although many studies regarding “diagnostic labelling” primarily address ASD or ADHD,^14^, ^15^, ^16^ a subset of studies explore a reconceptualization of TS. This moves away from a traditional emphasis on tic reduction toward a “letting be” approach, which prioritizes acceptance and accommodation of tics as part of an individual's neurodiverse experience.^17^, ^18^

Overall, these studies often focus on health care professionals' perspectives, lacking an exploration of whether patients, caregivers, and health care professionals share similar perspectives on labelling TS.

## Aims and Objectives

This study aims to investigate the views of patients, caregivers, and health care professionals regarding current Tourette's syndrome (TS) labeling and other patient‐centric alternatives.

## Patients and Methods

We designed a pilot study with three distinct virtual and anonymous surveys targeting three different groups of respondents: patients, caregivers, and health care professionals (Data [Link], [Link]). The surveys were opened simultaneously and remained active for 3 months, from March 2024 to May 2024.

The surveys for patients and caregivers comprised 7 questions. The first three questions focused on the patient's demographics, such as age, age of diagnosis, and current location. This was followed by an open question about their interpretation of the TS label and a closed question to rate their sentiment toward it on a satisfaction scale from 0 to 5, with 0 being not satisfied at all and 5 being completely satisfied. Additionally, respondents were presented with a list of 10 alternative terms for TS and asked to indicate their preference (single answer). An open‐ended section allowed participants to share any additional comments. Caregivers expressed their views in the second part of the survey. Quantitative data were subjected to statistical analysis and qualitative data to thematic analysis.

The health care professional survey comprised 6 questions, including geographical location and clinical role. The second part of the survey was identical to the caregivers and patient questionnaires.

We disseminated these surveys through TS networks, including the ESSTS and MDS TS Study Groups. They were supplied with QR codes, which the participants could quickly scan.

## Results

This initial pilot study received 421 responses from 26 countries, including 174 patients, 160 caregivers, and 89 health care professionals, of whom 38% have an expertise in neuropsychiatry. Of these, 242 responses were from the UK, and between 40 and 60 responses each from Germany, the USA, and Australia, with the remaining responses (<20) from Italy, Mexico, Nigeria, France, and Canada. Most patients who completed the survey were aged 18–24 years (24%), with caregivers/parents filling in the form for younger patients. Sixty percent of the respondents were diagnosed between the ages 0 and 17 years, and 27% were diagnosed between 18 and 34 years.

Overall, patients and caregivers were most satisfied with the term TS, with an average rating of 3.79/5 and 3.74/5, respectively.

Health care professionals rated the term slightly lower (3.11/5).

Of the 61 additional comments provided by the patients, 82% expressed concern about changing the label due to a sense of identity and community. These individuals felt that other terms, such as neurodiversity or tic disorder, would not be specific enough. However, some health care professionals and caregivers emphasized the negative stigma associated with TS through the comments. Altogether, 72% of health care professionals and 48% of caregivers favored an alternative term.

**FIG. 1:**
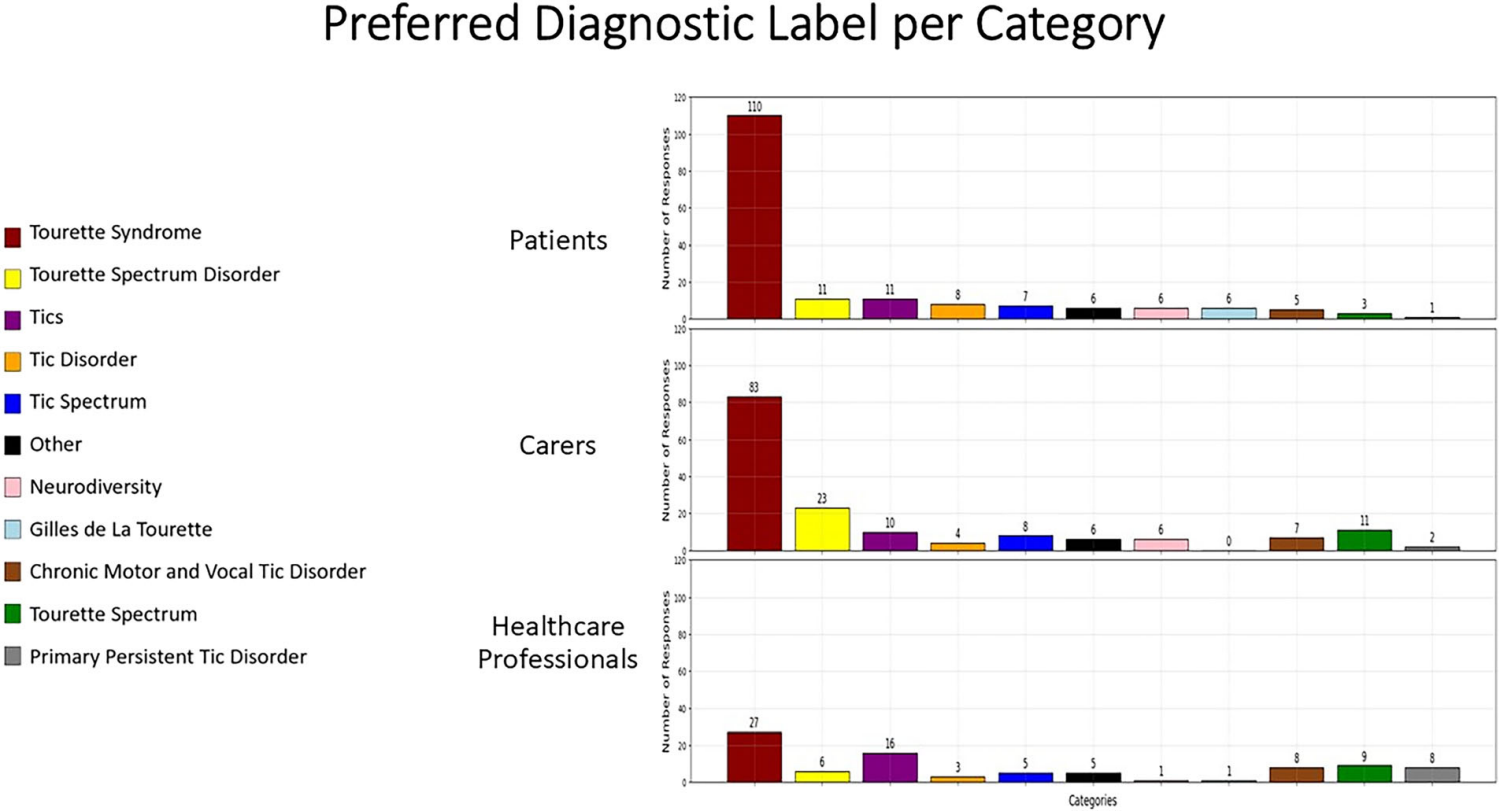
Preferred diagnostic label per category. This figure illustrates the preferred diagnostic label for Tourette's syndrome (TS) among patients, caregivers, and health care professionals. The graphs show a marked preference for the term TS across all groups.

Most patients (62.9%) and caregivers (51.4%) preferred the name TS over proposed alternatives compared to 29.3% of health care professionals (Fig. 1).

Within the free‐text answers, 3 main themes have been identified:

1. The importance of an accurate TS diagnosis and access to support
2. The need for lived experience‐centered language and understanding
3. The TS label as a community, identity, and empowerment

Following are some of the comments left by the respondents:

- *Please be guided by people with Tourette Syndrome and their families and carers. Tourette Syndrome is a difficult diagnosis to live with; it is so much more than tics. If you are looking to improve the lives of people with Tourette Syndrome, then there is a lot of scope for advocating for people to understand more about the support needed by people with Tourette Syndrome and reducing stigma*.
- *Tourette Syndrome is the word we have, and [we] have built knowledge, community, and identity around it*.
- *If your symptoms lean towards Tourette Syndrome, then you should be diagnosed as such, as not having a true diagnosis causes difficulties accessing the correct support and treatments. I do not see my diagnosis as a label, but as a reason for the way I am as a person*.
- *Getting a Tourette's Diagnosis for me has been crucial. It finally explained the tics I have had since I was very young. It has given me information, vocabulary, and a community of similar people*.

## Discussion

These survey results indicate that patients and caregivers strongly favor the diagnostic term “Tourette syndrome.” In contrast, some clinicians preferred the term *“Tourette spectrum*.” In addition, patients reported concerns regarding the implications of redefining TS. These concerns often centered on the fear of losing established communities, resources, and the shared identity built around TS.

It is important to emphasize that addressing the concerns of patients and caregivers by reassuring them about the continued availability of resources and the stability of their communities could ease these worries and lead to different outcomes.

## Diagnostic Labeling and Its Significance

Despite some of the neurodiversity movement's view of autism as a difference rather than as a disorder, this paper highlights that many patients value and feel that the TS diagnostic label is essential. Society currently requires labels to access support, as they can facilitate access to appropriate support and aid in self‐understanding and identity formation.^19^

The transition to a new diagnostic label for TS, similar to the reclassification of Asperger's disorder as ASD, could present significant practical challenges across multiple domains. This shift may disrupt ICD coding systems, complicate processes for health insurers, and impact research priorities and funding allocations. The transition from Asperger's disorder to ASD illustrates how diagnostic reclassifications necessitate extensive adaptations within medical, educational, and research frameworks.^20^ A formal diagnosis can be necessary to aid access to support within educational settings or the workplace. For instance, a diagnosis of TS can provide education modification, supportive teaching styles, or extra time in exams. In the workplace, it can enable adjustments to the environment or expectations, creating a more supportive environment. Individuals may struggle to receive assistance and understanding without a label.

Diagnostic labels can also support self‐understanding and identity, which the survey respondents raised. They provide a framework for understanding their experiences, strengths, and challenges.

Additionally, labels can play a significant role in advocacy efforts. They allow individuals and groups to rally for rights, accommodations, and recognition, often highlighting the need for societal changes to accommodate neurological differences.

Accurate labels are needed to promote research and understanding. This improves the development of novel therapies and helps inform social policies, as well as prevents misdiagnosis and inappropriate treatment. There have also been challenges with the confusion around multiple labels, for example, the differentiation of primary TS tics and functional tic‐like behaviors (FTLB) and the difficulties when both are present in the same individual.^21^ Accurate terminology helps to ensure that interventions are appropriately tailored to the individual's neurological profile.

## Limitations

This preliminary study encountered several limitations. A primary limitation is the lack of control over the authenticity of the respondents. Because our survey was open to everyone and shared online, we could not guarantee that the sample represented solely TS patients, caregivers, or health care professionals but could also include FTLB patients. However, we believe this limitation's impact can be minimized due to the substantial number of responses received and the primary distribution method from clinicians to patients and caregivers in the clinic.

Another limitation is a potential sampling bias. Given the controversy, participants who left negative comments may have had firm opinions, possibly influenced by an earlier diagnosis. To address this, we will focus on the perspectives of newly diagnosed patients (<12 months since diagnosis) in the next study. Nonetheless, the diverse demographic and age distribution of the current survey helps mitigate this limitation by providing a wide range of views.

Lastly, as a project conducted solely online, another limitation is the low response rate from individuals in remote areas or those with less access to technology. In the next steps, we aim to reach remote groups by distributing paper versions of the surveys through wider networks. Nonetheless, the online survey allowed us to include non‐English‐speaking countries through real‐time translation.

## Conclusions and Future Work

“Tourette syndrome” is a historical name that appears to be the preferred label. This study highlights the importance of directly involving patients in discussions on diagnostic labeling and terminology.

To broaden our understanding within the TS community and specialized TS health care professionals, we plan to perform a further wide‐scale project.

The new surveys include questions about sex, ethnicity, geographical location, and role of health care professionals, with an exclusion criterion of age <8 years old. A specific threshold of a preference for an alternative label for >50% of patients and carers will be used as a base to advocate for a new label.

In future work, the concerns of patients regarding the continuity of support and services will be carefully considered. We will evaluate whether these concerns influence their perspectives on diagnostic labelling.

We will canvas views from several countries, with planned involvement from partner organizations, including the TTAG (Tics and Tourette Across the Globe), MDS, and ESSTS communities.

## Author Roles

(1) Research project: A. Conception, B. Organization, C. Execution; (2) Statistical analysis: A. Design, B. Execution, C. Review and critique; (3) Manuscript: A. Writing of the first draft, B. Review and critique.

G.R.: 1A, 1B, 1C, 2A, 2B, 2C, 3A, 3B

O.M.: 1C, 2C, 3B

S.A.: 1C, 2C, 3B

L.C.: 1C, 2C, 3B

A.H.: 1C, 2C, 3B

D.M.: 1C, 2C, 3B

N.M.D.: 1C, 2C, 3B

K.R.M: 1C, 2C, 3B

C.N.: 1C, 2C, 3B

T.P.: 1C, 2C, 3B

S.S.: 1C, 2C, 3B

N.S.: 1C, 2C, 3B

O.T.: 1C, 2C, 3B

K.K.T.: 1C, 2C, 3B

T.H.: 1A, 1B, 1C, 2A, 2B, 2C, 3A, 3B

## Disclosures

**Ethical Compliance Statement**: The authors confirm that the approval of an institutional review board was not required for this work. Informed consent was obtained from all the participants of this study. We confirm that we have read the journal's position on issues involved in ethical publication and affirm that this work is consistent with those guidelines.

**Funding Sources and Conflicts of Interest**: No specific funding was received for this work.

**Financial Disclosures for the Previous 12 Months**: Kirsten Müller‐Vahl has received financial or material research support from DFG: GZ MU 1527/3‐1 and GZ MU 1527/3‐2 and Almirall Hermal GmbH. She has received consultants' and other honoraria from AlphaSights Ltd., Canopy, Canymed, DHMS Direct Health Medical Services Ltd./ Wellster Healthtech Group, Emalex, Eurox Group, Neuraxpharm, Sanity Group, Stadapharm GmbH, Swiss alpinapharm, Synendos Therapeutics AG, Tetrapharm, and Triaspharm. She is an advisory/scientific board member for Branchenverband Cannabiswirtschaft e.V. (BvCW), Sanity Group, Synendos Therapeutics AG, Syqe Medical Ltd., and Therapix Biosciences Ltd. She has received speaker's fees from Almirall, Bundesverband der pharmazeutischen Cannabinoidunternehmen (BPC), Cogitando GmbH, Emalex, Grow, Laleto GmbH, Landesamt für Soziales, Jugend und Versorgung Mainz, Medizinischer Dienst Westfalen Lippe, Noema, streamedup! GmbH, VBG—Unfallversicherung Hamburg, Vidal, and WeCann. She has received royalties from Elsevier, Medizinisch Wissenschaftliche Verlagsgesellschaft Berlin, and Kohlhammer. She is an associate editor for “Cannabis and Cannabinoid Research.” She is an editorial board member of “Medical Cannabis and Cannabinoids” and “MDPI‐Reports” and a scientific board member for “Zeitschrift für Allgemeinmedizin.”

## Supporting information


**Data S1.** Supplementary material for review and publication 1: caregiver survey on Tourette's syndrome (TS) label preference. This survey was administered to caregivers and/or parents of individuals with TS. It includes questions regarding their preferred diagnostic label for the condition.

**Data S2.** Supplementary material for review and publication 2: health care professional survey on Tourette syndrome label preference. This survey was completed by health care professionals, some of whom diagnose and/or treat individuals with TS. It explores their preferred terminology when referring to the condition and their perspectives on different labels.

**Data S3.** Supplementary material for review and publication 3: patient survey on Tourette's syndrome (TS) label preference. This survey was distributed to individuals diagnosed with TS. It enquires on their preferred diagnostic label and how different terms impact their perception of the condition.

## Data Availability

The data that support the findings of this study are available from the corresponding author upon reasonable request.
