# Supplementary figures and images for: Tic‐Talk: Voices on Tourette's Labelling
Source: Mov Disord Clin Pract. 2025 May 2;12(7):917–21. doi: 10.1002/mdc3.70110 (PMC12274994; doi:10.1002/mdc3.70110)

# “Tic-Talk: Voices on Tourette Labelling” survey distributed to caregivers and parents.

#
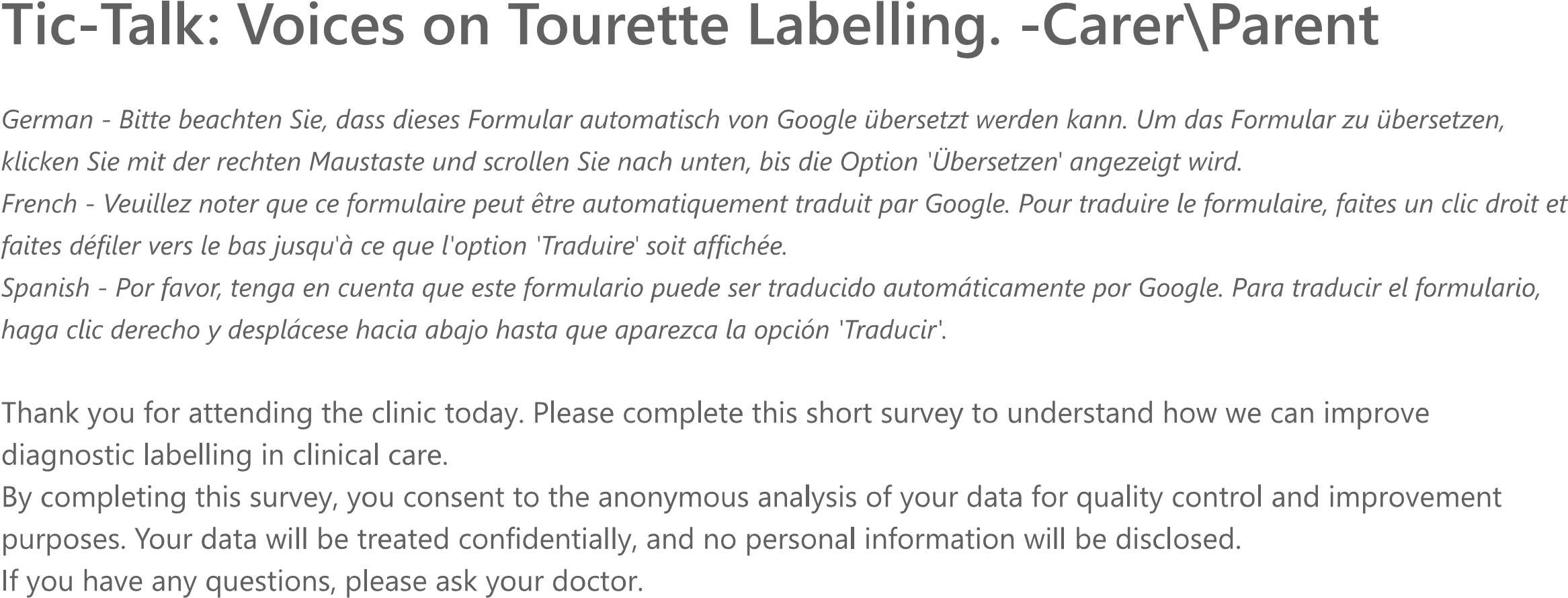


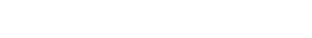

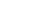

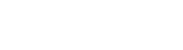

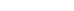


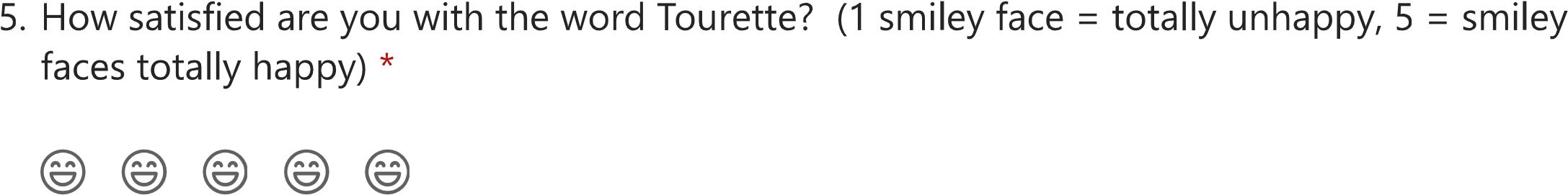


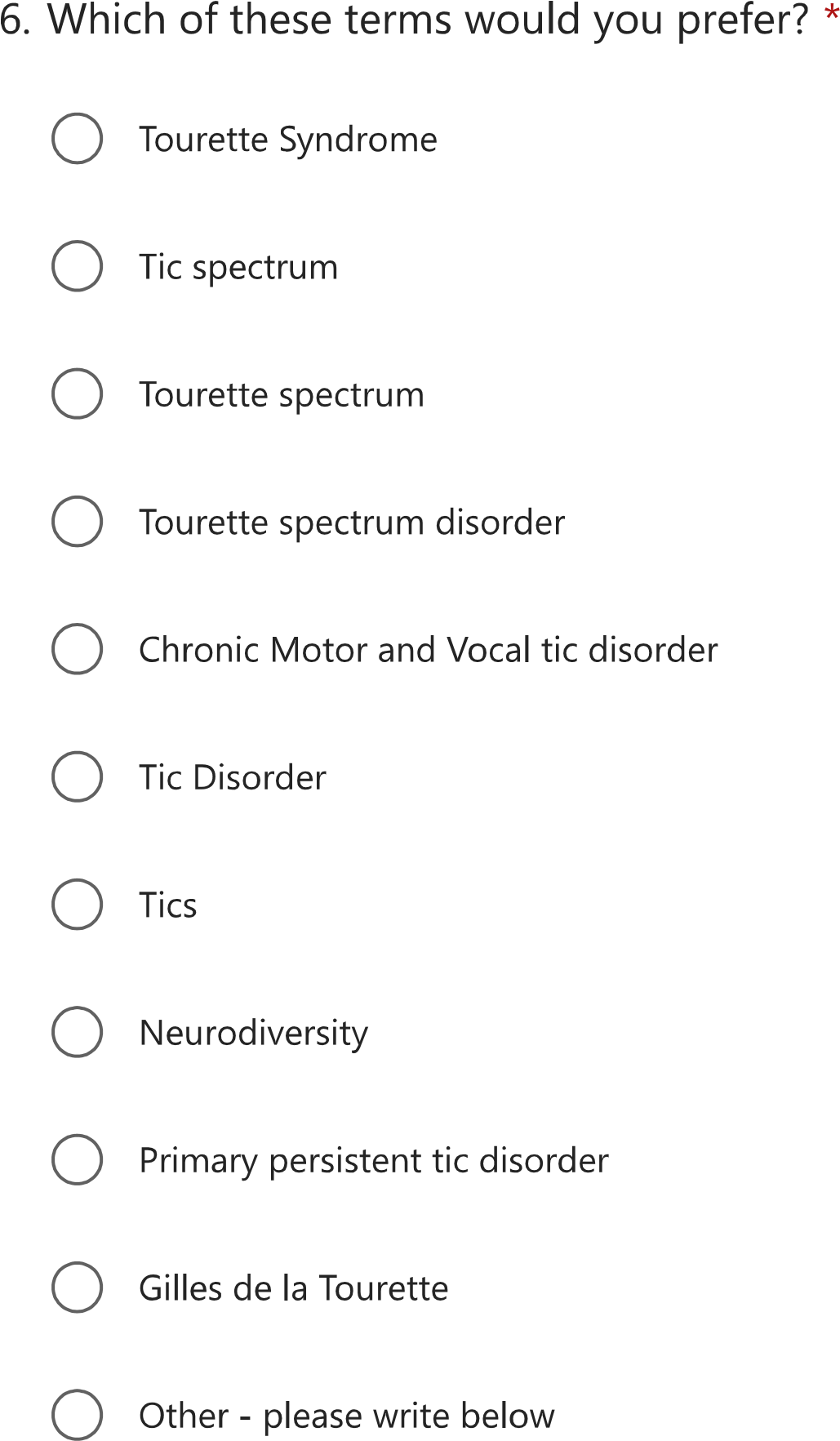

Supplement: Supplementary file 1 — Data S1. Supplementary material for review and publication 1: caregiver survey on Tourette's syndrome (TS) label preference. This survey was administered to caregivers and/or parents of individuals with TS. It includes questions regarding their preferred diagnostic label for the condition. [file MDC3-12-917-s003.docx]

# “Tic-Talk: Voices on Tourette Labelling” survey distributed to healthcare professionals.


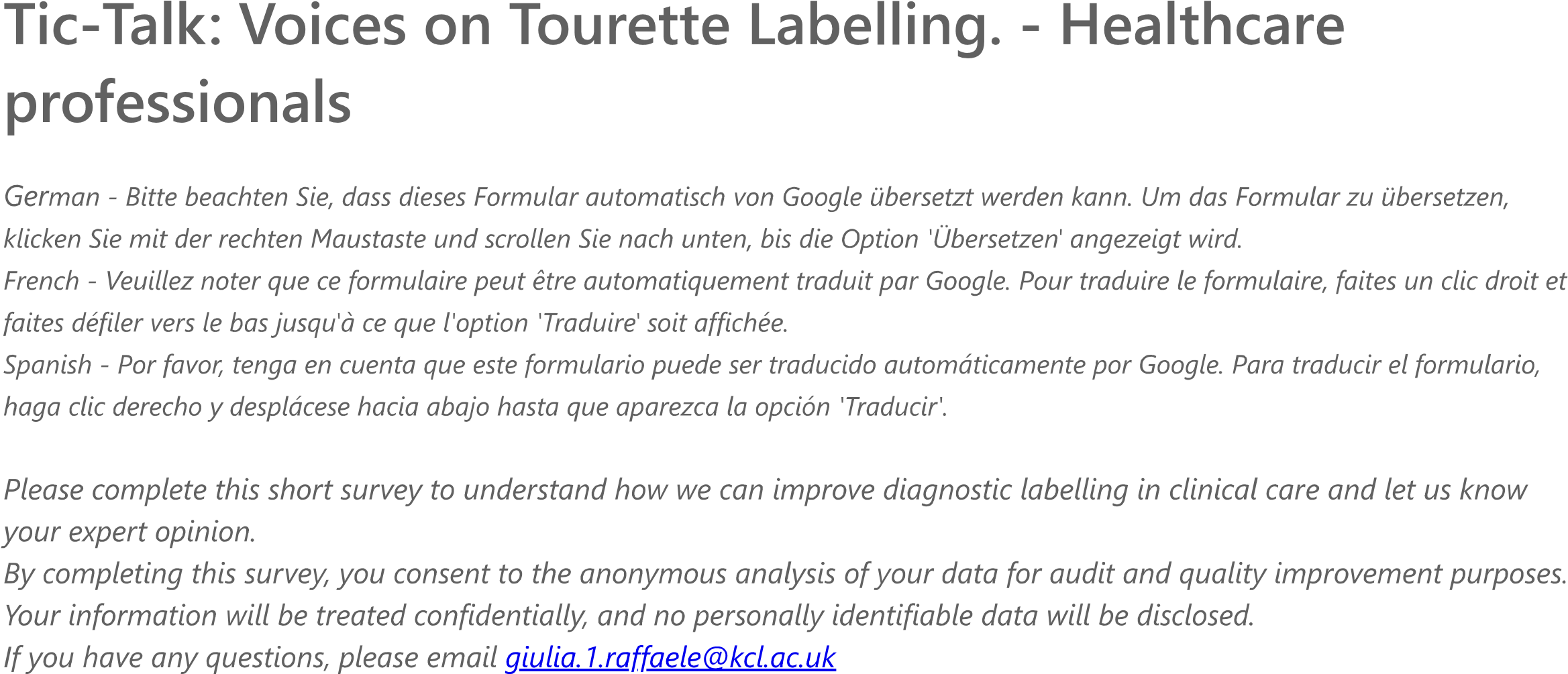


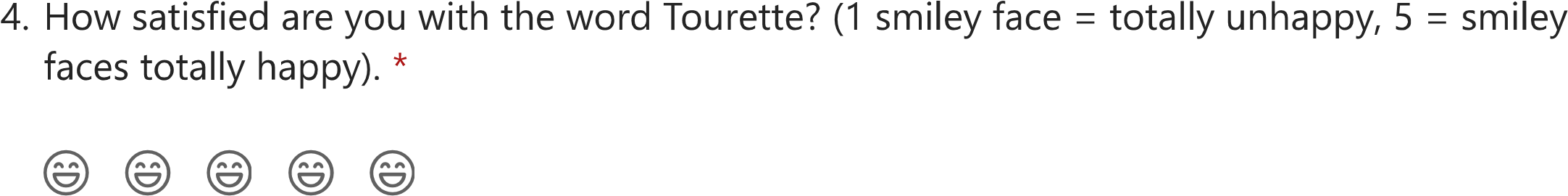


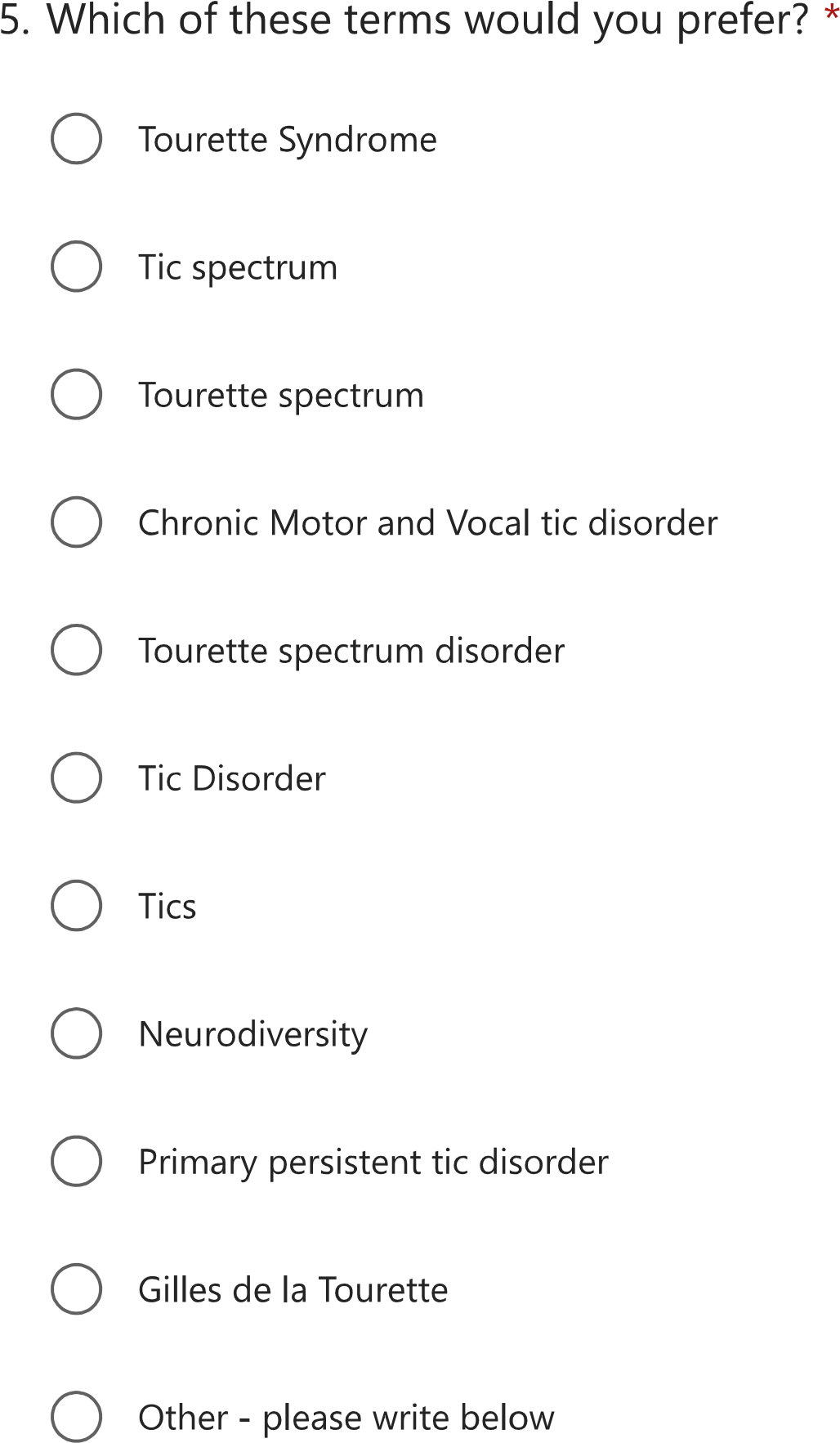

Supplement: Supplementary file 2 — Data S2. Supplementary material for review and publication 2: health care professional survey on Tourette syndrome label preference. This survey was completed by health care professionals, some of whom diagnose and/or treat individuals with TS. It explores their preferred terminology when referring to the condition and their perspectives on different labels. [file MDC3-12-917-s001.docx]

# “Tic-Talk: Voices on Tourette Labelling” survey distributed to patients.


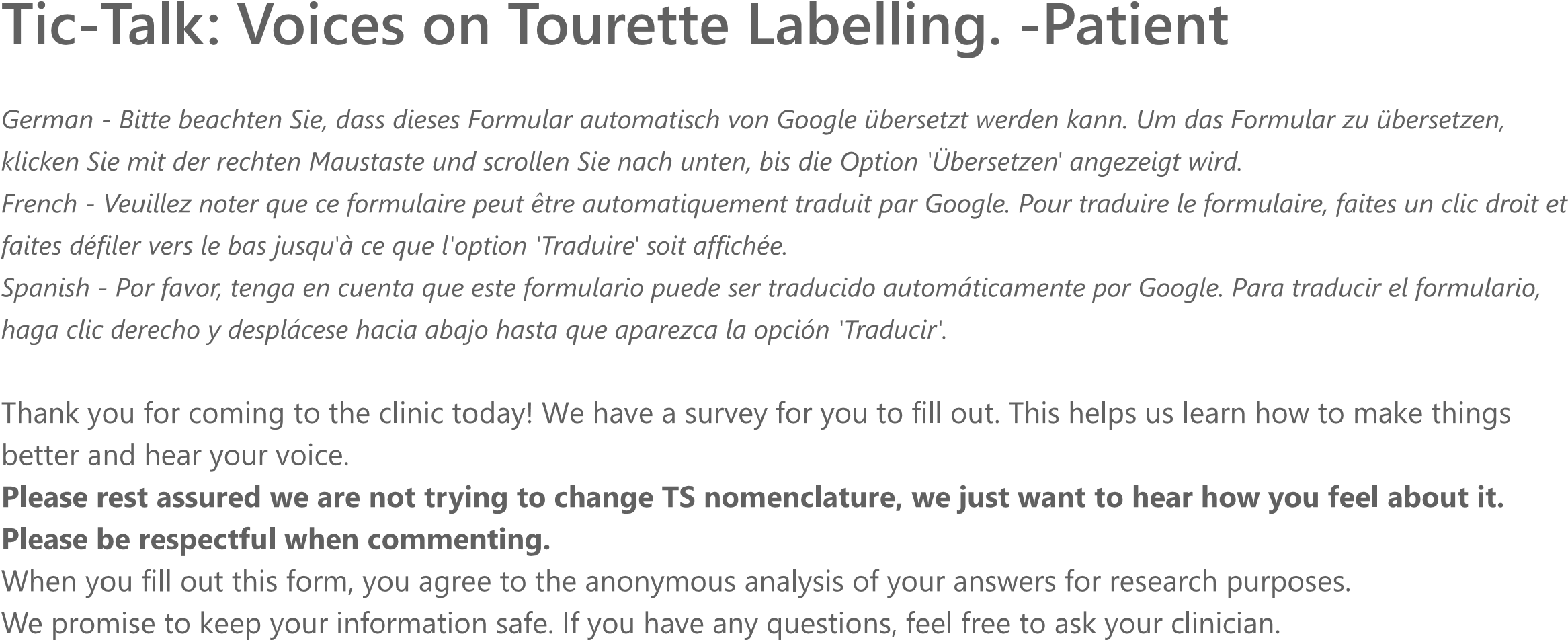


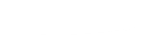

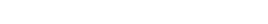


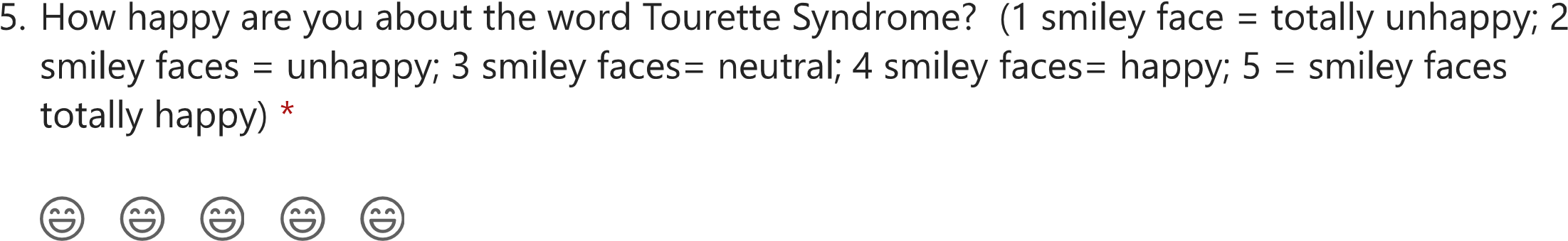


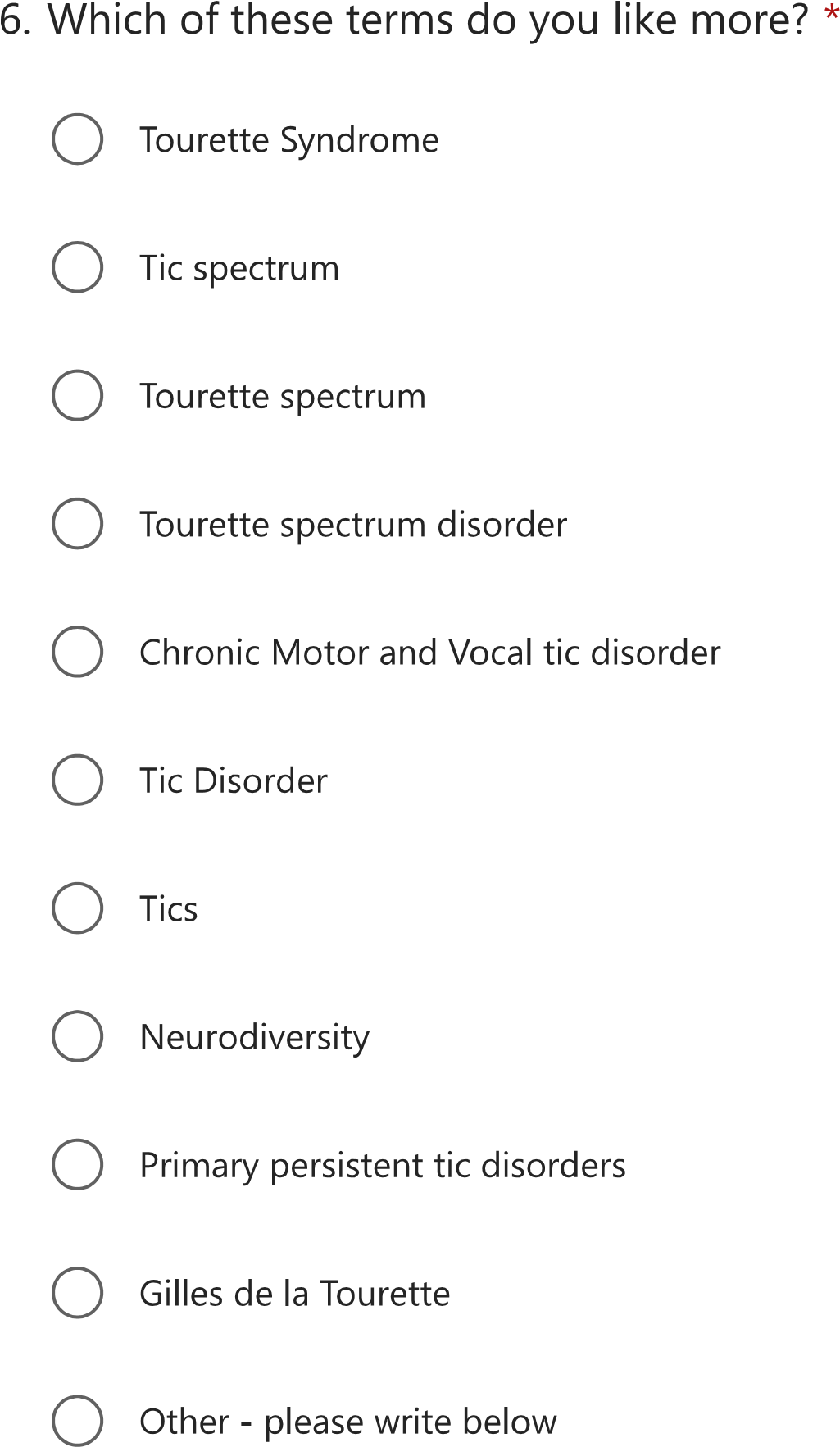

Supplement: Supplementary file 3 — Data S3. Supplementary material for review and publication 3: patient survey on Tourette's syndrome (TS) label preference. This survey was distributed to individuals diagnosed with TS. It enquires on their preferred diagnostic label and how different terms impact their perception of the condition. [file MDC3-12-917-s002.docx]
